# Effect of community-based group exercises combined with action observation on physical and cognitive performance in older adults during the Covid-19 pandemic: A randomized controlled trial

**DOI:** 10.1371/journal.pone.0295057

**Published:** 2023-12-05

**Authors:** Bagdat Tekkus, Fatma Mutluay

**Affiliations:** 1 Department of Physiotherapy and Rehabilitation, Institute of Health Sciences, Istanbul Medipol University, Istanbul, Turkey; 2 Institute of Health Sciences, Istanbul Medipol University, Istanbul, Turkey; UFPE: Universidade Federal de Pernambuco, BRAZIL

## Abstract

**Objective:**

This study investigates the impact of community-based exercises with action observation therapy (AOT) on the physical and cognitive performance of older adults experiencing social isolation during the COVID-19 pandemic.

**Methods:**

One hundred participants aged 65–80 years were randomly divided into two groups: the AOT group, which engaged in balance, strengthening, and mobility exercises guided by 15-minute action observation videos before a 45-minute exercise session, and the control group, which performed the same exercises without action observation. Both groups underwent three sessions per week for eight weeks (24 sessions in total). The assessment tools used in this study included the following: For evaluating mobility and fall risk in older adults, the Timed Up-and-Go (TUG) Test was employed. To assess functional strength of lower extremities, balance, and fall risk, the Five Times Sit-to-Stand (5XSST) Test was administered. Balance and gait were measured using the Tinetti Balance and Gait Assessment (TBGA), utilizing the Tinetti Scale. Individuals’ confidence in performing daily activities without falling or losing balance was assessed using the Activities-Specific Balance Confidence Scale (ABC). Furthermore, cognitive functions across multiple domains, including attention-concentration, executive function, memory, language, visual construction skills, abstract thinking, calculation, and orientation, were evaluated using the Montreal Cognitive Assessment (MoCA) Tests.

**Results:**

Results revealed significant improvements in both groups. Group I, which received Action Observation Therapy (AOT) in addition to exercise, demonstrated superior outcomes in the 5XSit-to-Stand test (Δ = -1.92, p < 0.0001, Cohen’s d = 0.77), Tinetti Balance and Gait Scale (Balance: Δ = 2.77, p < 0.0001, Cohen’s d = 0.91), and Timed Up and Go test (Δ = -1.98, p < 0.0005, Cohen’s d = 0.83). On the other hand, Group II, which received exercise only, exhibited substantial gains in the Tinetti Balance and Gait Scale (Walking: Δ = 0.52, p < 0.01, Cohen’s d = 0.27) and Activity-Specific Balance Confidence Scale (Δ = 5.77, p < 0.0001, Cohen’s d = 0.26).

**Conclusion:**

These findings underscore the effectiveness of AOT-enhanced community-based exercises in enhancing both physical and cognitive performance among older adults facing social isolation during the pandemic, with Group I (AOT + exercise) showing particularly promising results.

**Trial registration:**

This study was registered with ClinicalTrials.gov Identifier: NCT04759690, ClinicalTrials Protocol ID: p3957ghb.

## Introduction

During the Covid-19 pandemic period, physical, social and mental health were affected all over the world [1, 2]. The protective measures and lockdown restrictions applied during the pandemic increased sedentary behavior, adversely affecting participation in physical activity. Tison [3] reported that the average number of steps per day decreased even in countries such as South Korea, Taiwan, and Japan with relatively low COVID-19 infection rates who did not institute lockdowns. In older adults in particular, who have been significantly more and sharply affected [2, 4], the risk of decreased functionality, loss of physical performance, balance problems and injuries due to falls, which are already seen due to aging, can be expected to increase with sedentary lifestyle. In this context, interventions aimed at protecting and improving individual health in the older adult population by maintaining physical activities, and especially those performed outdoors, have been strongly recommended even during the pandemic period [1, 5].

Community-based multi-component interventions combining motor, sensory and cognitive rehabilitation techniques are frequently used for health promotion. Their benefits to physical health have been shown for older adults [1, 6, 7]. They are also cost advantageous when easily accessible areas such as public meeting places or parks are available.

In recent years, cognitive strategies including Action Observation Therapy (AOT) have begun to be combined with rehabilitation programs to enhance motor learning and functional recovery in patients with both neurological and musculoskeletal disorders [8–10]. Action observation is a cognitive process based on the individual observing the actions performed by others without any movement execution. In AOT practice, participants first observe a meaningful action and then mimic it themselves. Thus, motor learning and motor memory formation are stimulated and it becomes easier to understand and imitate movements [11–13]. AOT is a complementary strategy used to develop motor skills, facilitate motor learning, and stimulate neuroplasticity [8–10]. Neurophysiology studies have suggested AOT stimulates the activity of mirror neuron systems associated with motor and motor-related neural networks [11, 12, 14]. AOT has been shown to improve upper extremity motor recovery, activities of daily living, balance and walking in neurological diseases like stroke [15–18], Parkinson’s disease [19–21], cerebral palsy [22, 23] and to have significant beneficial effects on physical function and walking in orthopedic patients undergoing surgery [24–27]. AOT has also been reported to enhance the effectiveness of exercises for improving postural control, gait parameters, static and dynamic balance in healthy youth [28] and to reduce the risk of falling in older adults [29, 30]. Considering the demonstrated beneficial effect of AOT for improving motor performance by exercising, one should expect it should also benefit older adults to improve their motor functions which have been impaired by the pandemic restrictions.

The purpose of this study was to assess the effects of a specially designed multi-component outdoor community-based exercise program on physical and cognitive performance of older adults exposed to physical inactivity induced by the Covid-19 pandemic. The possible added benefit obtained by combining Action Observation Therapy with this program has also been investigated.

## Materials and methods

### Study design

This study was designed as a single-blind, randomized controlled trial. The study protocol was approved by the İstanbul Medipol University, Non-interventional Clinical Studies Ethics Committee with the date of October 2020. (Reference:10840098–772.02-E.58328). In accordance with the principles of the Declaration of Helsinki, all volunteer participants were informed about the study and their written consent was obtained before entering the study. This study was registered with ClinicalTrials.gov Identifier:NCT04759690. The study was carried out between December 2020 and December 2021.

### Participants and sample

The study targeted older adults aged 65–80 years living in Abbasağa district of Beşiktaş municipality in İstanbul, Turkiye. It was carried out between December 2020 and December 2021 during the period of partial restrictions of the Covid-19 pandemic by obtaining the permission of the local municipality. The restrictions in place forbade all outdoor activities to persons 65 and older except only at 10:00–14:00 on weekdays.

A systematic filtering of the municipal registry for the targeted population yielded a list of 2552 persons. With a probabilistic random sampling method, every 12th person on this list was called by phone; 200 people could be successfully contacted and invited to the study. 100 persons accepted and were invited to the local municipal health clinic where they were individually interviewed and tested for the study criteria in a quiet and spacious room. The inclusion criteria were: a) being between 65–80 years old b) getting a score of 24 or higher on the Mini Mental State Examination c) not having visual or auditory problems d) being able to walk without using assistive devices e) not having neurological or musculoskeletal disorders that prevent exercise. Exclusion criteria were: a) uncontrolled hypertension and diabetes mellitus b) chronic obstructive pulmonary disease c) cardiovascular and cerebrovascular disease or a history of traumatic brain injury d) major surgery in the past 6 months that causes walking difficulties.

The demographic and clinical characteristics of 60 candidates meeting the study criteria were recorded during the individual interviews. Each of them executed an individual trial exercise session under physiotherapist supervision in the same room after which the individual’s self-reported fatigue level according to the Modified Borg Scale (1–10) was noted [31]. The physiotherapist guided, monitored and supervised each individual exercise performance and answered participants’ questions.

### Groups and interventions

Prior to the randomization process, participants did not engage in any exercise sessions to ensure the impartiality of the researcher regarding their exercise capacity. The decision was made to maintain confidentiality regarding participants’ exercise capabilities. However, after random assignment to either the experimental or control group, participants underwent the following sequence of steps:

#### Randomization

Participants were randomly assigned to either the experimental (AOT + exercise) or control (exercise only) group. The randomization process for participant selection was executed with precision. Following the initial contact of 200 individuals who had agreed to participate, a meticulous assessment against the study criteria was conducted in a tranquil and spacious environment at the local municipal health clinic. The inclusion criteria encompassed individuals aged 65 to 80 years, scoring 24 or higher on the Mini Mental State Examination, without visual or auditory impairments, capable of walking unassisted, and devoid of neurological or musculoskeletal conditions impeding exercise. Exclusion criteria comprised uncontrolled hypertension, diabetes mellitus, chronic obstructive pulmonary disease, cardiovascular and cerebrovascular ailments, a history of traumatic brain injury, and major surgery within the preceding 6 months that resulted in walking difficulties. This comprehensive selection process ensured the integrity and appropriateness of participants in the study, maintaining rigorous research standards.

#### Group observation

Once assigned to their respective groups, participants were observed within their groups. Importantly, the experimental and control groups conducted their exercise sessions on different days and at different times to prevent any potential influence or interaction between the two groups.

#### Independent observers

The study employed independent individuals for various roles to maintain objectivity. Different individuals were responsible for randomizing participants, observing the experimental and control groups during their exercise sessions, and conducting evaluations.

Participants (n = 60) were randomized using sealed opaque envelopes blindly selected by an uninvolved third party into two groups: Group-I (Action Observation Therapy and Exercise: AOT+E, n = 30) and Group-II (Exercise only: E, n = 30). Each group was further subdivided into training subgroups of 10 persons with similar recorded individual Borg fatigue scores. Initial training schedules, subject to revision due to weather and other unforeseen factors, were prepared and communicated in written form to each participant.

### Exercise program

In light of the COVID-19 pandemic, all pre-test and post-test assessments were conducted in indoor, face-to-face settings, with strict adherence to hygiene protocols, including the use of masks and maintaining a physical distance of 3 meters between participants and assessors. This approach ensured the safety and hygiene of all participants while facilitating the accurate collection of data.

#### Safety measures and infection prevention

The study prioritized participant safety amidst the COVID-19 pandemic by implementing rigorous safety measures. Participants in both the experimental and control groups were required to wear masks during exercise sessions to minimize the potential transmission of the virus. Additionally, to adhere to social distancing guidelines, participants were strategically positioned during exercises, with one individual positioned slightly forward and another placed to the side and further back, ensuring adequate spacing between participants. Furthermore, all exercise equipment used by elderly participants underwent thorough cleaning and sanitization before and after each session to maintain a hygienic environment. These precautionary measures were diligently observed to mitigate infection risks and safeguard the well-being of all volunteers, contributing to the integrity of the study’s results and facilitating future research planning during pandemics.

Participants lived in an upper middle class metropolitan urban district with a strong majority of appartment flats. Exercise sessions were conducted in a wooded park centrally located in this district within easy, less than 10 minutes walking distance from the participants home locations. This park contained flat, amenaged areas suitable for group exercises. Participants were requested to wear confortable clothes and sports shoes. All exercise related equipment such as therabands, sandbag weights was provided by the supervising physiotherapist at no cost to the participants.

All subgroups performed the outdoor exercises as a group activity under the supervision and control of an experienced physiotherapist, 3 days a week for a total of 8 weeks (24 sessions) between 10:15–13:45 in fair weather conditions following social distancing rules in accordance with current regional pandemic conditions. Group-I and Group-II subgroup training sessions were scheduled in separate days to blind the participants to AOT application. In order to ensure their safety, optional chair support was provided to each participant during the exercises.

A 15-minute action observation video including balance, strength and mobility exercises was prepared by and modelled in by the researcher using a chair to guide those who might need its usage during the exercises. Each exercise session in both the experimental (Group I) and control (Group II) groups lasted approximately 45 minutes.

#### Exercise program description

The rehabilitation program employed in this study consisted of a carefully designed set of exercises aimed at enhancing strength, balance, and mobility in older adults. These exercises were chosen in alignment with the American College of Sports Medicine (ACSM) guidelines for individuals aged 65 and older. A 15-minute action observation video was created, illustrating these exercises, allowing participants to observe and learn from the video content. The exercises progressed from simple to more complex movements, from static to dynamic, and from exercises with a wide support base to those with a narrower base, ultimately incorporating walking exercises.

In the experimental group (Group I), participants watched this action observation video before each exercise session to familiarize themselves with the movements. Subsequently, they actively performed these exercises for 45 minutes under the guidance and supervision of a research physiotherapist. To clarify, the training sessions were conducted three times a week with one-day breaks between sessions. In contrast, the control group (Group II) performed the same exercises without the preceding action observation component. All participants received support from chairs during exercise sessions to ensure safety.

### Progression of exercises

The exercises progressed from simple to complex, static to dynamic, from wide support surface to narrow support surface and walking. Although our study was performed as a group exercise, the intensity of exercises was determined by taking individual differences into account. The initial exercise intensity of each subgroup was determined according to the minimal Borg Scale individual fatigue level reported in that subgroup during the trial sessions conducted earlier during the qualifying phase of the study. Target training Borg score started at 3–4 (light difficulty) and gradually progressed to 5–6 (medium difficulty). The exercises were started with 5 repetitions, gradually increasing to 8–10 repetitions according to improvements observed by the supervising physiotherapist during the later sessions.

#### Exercise program sources

The rehabilitation program utilized in this study, focusing on strength, balance, and mobility exercises tailored to individuals aged 65 and older, was developed based on guidelines recommended by the American College of Sports Medicine (ACSM) [32]. This program was structured and prepared by the researchers, taking into account the specific needs of older adults. While the program draws its foundation from ACSM guidelines, it was further customized to meet the unique requirements of the study participants.

### Exercise sessions

Group-I participants watched the action observation video for 15 minutes on a 45-inch screen connected to a personal computer before exercising in each session and they were asked to mimic these movements in the following period where they actively performed these exercises themselves under physiotherapist supervision. Group-II participants did the same exercises without any such previous action observation with physotherapist guidance.

Training exercises were created from simple movements that are easy to learn, suitable for participants over 65 years of age. They included balance, strengthening and mobility exercises which are detailed in Table 1. Each session was preceded with a five minute warm-up which was followed by active exercise practice and concluded with a five minutes long cool-down period. Sessions lasted approximately one hour.

**Table 1:** Training programs for with action observation (AOT: Group-I) and simple exercise (E: Group-II) groups.

Action Observation (15 min)	Warm-up (5 min)	Community-based exercise (45 min)	Cool-down (5 min)
**Group-I only:**	**Sitting:**	**Sitting (strengthening):**	**Sitting:**
The video shows the model doing the balance, strength, and mobility exercises.	• Neck flexion, extension and rotation	• Shoulder flexion, abduction, horizontal abduction and adduction (with TheraBand)	• Neck flexion, extension and rotation
	• Shoulder elevation and circumduction		• Shoulder elevation and circumduction
	• Swinging leg forward and backward	• Knee extension (with sandbag)	• Swinging leg forward and backward
	• Dorsi flexion and plantar flexion	• Hip flexion (with sandbag)	• Dorsi flexion and plantar flexion
	**Standing:**	• Sit-to stand	**Standing:**
	• Stepping in place with arm swings	**Standing (balance and strengthening):**	• Stepping exercise with arm swings
		• Weight transfer to right and left legs	• Stretching (hamstring and gastrocnemius)
		• Stand on one leg (right-left)	
		• Heel and toe stand	
		• Tandem stance (right-left)	
		• Stepping in place	
		• Semi-squat	
		• Hip abduction and adduction	
		• Hip extension	
		**Mobility:**	
		• Free walking (forward and backward)	
		• Side walking	
		• Tandem walking (forward and backward)	

*Note*. Progression of strengthening exercises: First week: 5 reps. without resistance; Second week: 1 set and 5 reps. with resistance; Third week: 8 reps, 1 set with resistance; 4.-6. weeks: 8 reps. 2 sets with resistance; 7.-8. weeks:10 reps. 2 sets with resistance

### Evaluations

This study implemented a single-blind design to minimize bias and ensure the impartiality of the results. Blinding occurred at various stages of the research process. Firstly, the randomization process, conducted by a separate researcher, ensured that participants were assigned to either the experimental or control group without any knowledge of their group allocation. Secondly, during the exercise sessions, a different researcher served as the observer and guide for both groups, ensuring that they remained unaware of their group status. Furthermore, data evaluation and analysis were carried out by individuals who were unaware of which group the participants belonged to, as participants engaged in sessions on different days and times. This comprehensive blinding approach was adopted to maintain the integrity and objectivity of the study results.

**Fig 1:**
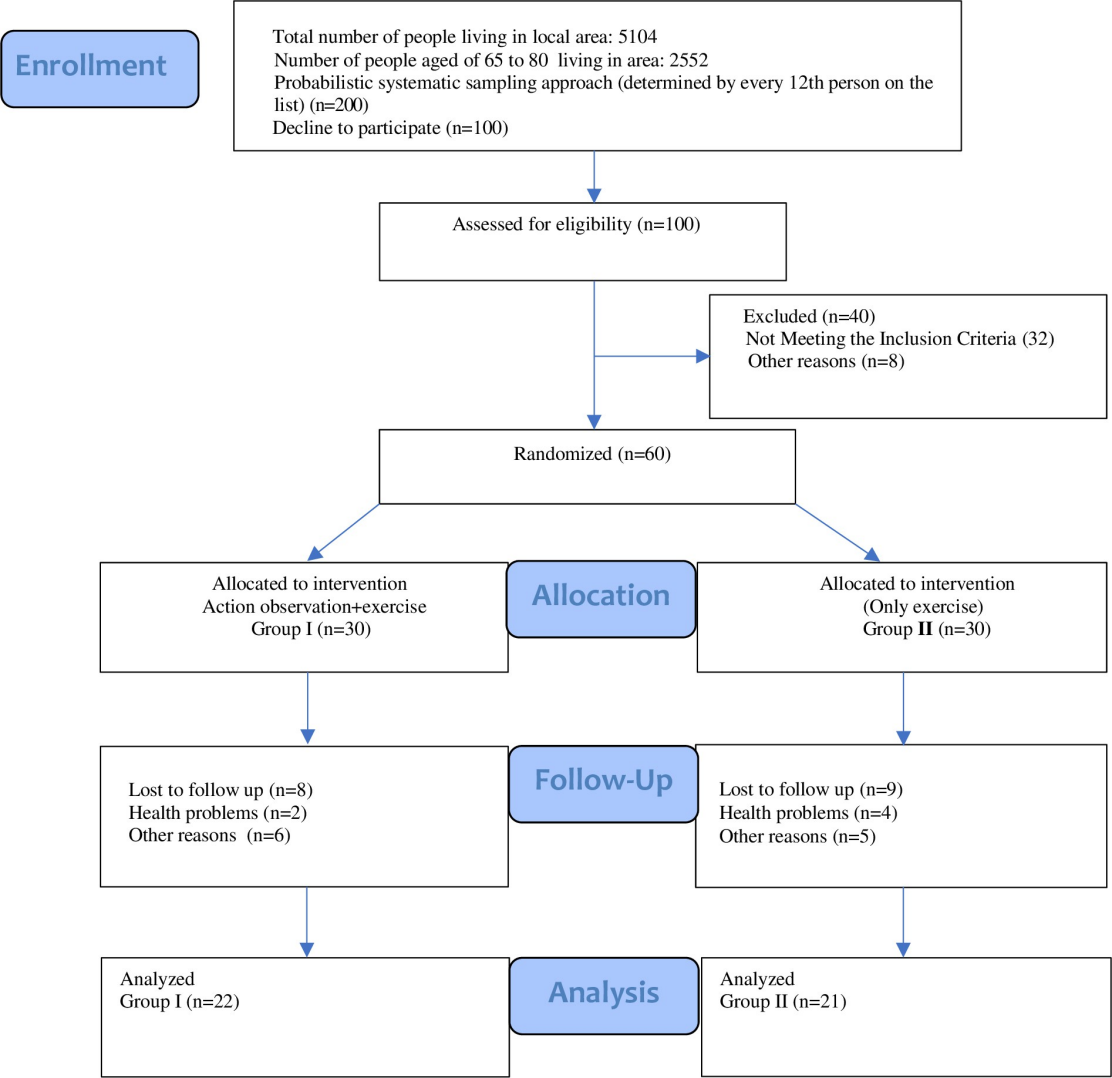
Flow diagram.

All data collection and pre-intervention outcome measure assessments were made by the researcher during the initial qualifying phase of the study participants in an isolated room at the local municipal health clinic. Outcome measurements were repeated at the end of the study at the same location. The detailed Consort flow chart of the study is given in Fig 1.

#### Demographic and clinical characteristics

Demographic information of participants including their age, sex, years of education, employement and marital status, smoking-alcohol use, as well as their adress and housing type was recorded. Additionally, participants selected the medical conditions they suffer from a list of common age related disorders (e.g., hypertension, diabetes, arthritis, hyperlipidemia) and reported their number of falling during the last year. Their height and weight were measured and their body mass index calculated [32].

#### Mini-Mental State Examination (MMSE)

It is a test that quantitatively evaluates the cognitive level (orientation, attention, calculation, close memory and language) globally. The highest possible total score is 30 and scores under 24 point to cognitive impairment [33]. Its adapted language version [34] was administered during the study inclusion criteria qualifying interview. The Mini-Mental State Examination (MMSE) has shown strong test-retest reliability, with reported values ranging from 0.80 to 0.95 in previous studies [35].

#### International Physical Activity Questionnaire ‐ Short Form (IPAQ-SF)

This is a 7-item questionnaire probing a person’s physical activities for at least 10 minutes at any time in the past seven days. The time spent (in minutes) on various physical activity levels are multiplied by their metabolic equivalent (MET) and summed to compute the physical activity category [36] in units of MET min/week: low (<600), moderate (600–3000), and high (>3000). The adapted language version of IPAQ-SF [37] was used at the beginning of the study to assess the activity category of the participants.

### Outcome measurements

The primary outcome of our study, as stated, is functional capacity assessed primarily through the Timed Up and Go (TUG) test, while global cognition measured by the MoCA serves as a secondary outcome.

#### Timed Up and Go (TUG) test

It is often used to assess the mobility and fall risk in aged people. The time (in seconds) it takes for the person to stand up from a chair (43–45 cm), walk a distance of three meters at normal speed and come back to sit on the chair is measured with a stopwatch. A shorter TUG time indicates better balance and mobility, while a testing time >12 seconds points to an increased risk of falling [38]. This test was selected as the primary outcome of this study due to its combined evaluation of mobility and balance, the existence of normative data as well as recent published results in a related study [30]. The Timed Up and Go (TUG) test demonstrated excellent test-retest reliability in various populations, including typical adults, individuals with cerebral palsy, multiple sclerosis, Huntington’s disease, stroke, and spinal cord injury, as reported in a review of 77 articles [39].

#### 5X Sit-to-Stand Test (5XSST)

This test assesses postural control and lower extremity muscle strength in relation to balance and falling risk. The participants are seated on a chair (43-45cm high) with their back straight and after crossing their arms over their shoulders, they are asked to get up and down five times as fast as they can, without stopping. The total time is measured with a stopwatch. There are normative values for this test according to age, with >12 second test time pointing to low functional strength and increased risk of falling for healthy older adults [40].

#### Tinetti Balance and Gait Assessment (TBGA)

It is a qualitative scale used to evaluate balance ability and gait in geriatric population. In the test questionnaire, 16 items consist of performance evaluation of movements made during activities of daily living (9 involving balance and 7 walking) accompanied by an observer [41]. The total maximum score is 28 (balance: 16 points, walking: 12 points); lower scores indicate higher risk of falling (≤18 points high, 19–23 moderate, ≥24 low risk). The adapted language version of TBGA was used in this study [42]. To ensure measurement reliability, the physothersapist guided a trial execution of TBGA questionnaire followed by its repeatition for the actual assessment of recorded TBGA score.

#### Activities-Specific Balance Confidence (ABC)

It is a 16-question questionnaire in which the individuals evaluate how much, as a percentage (%), they can confidently perform daily activities indoors and outdoors without falling and losing balance [43]. Each activity questioned is evaluated between 0–100 (0% = no confidence, 100% = full confidence); the highest total score which can be obtained by summing these evaluations is 1600. This total score is divided by 16 to yield the ABC score ranging from zero to 100%. An ABC score inferior to 60% is considered an indicator of low functional status in older adults. The adapted language version of ABC was used in the study [44].

#### Montreal Cognitive Assessment (MoCA)

It is a multi-domain screening test that evaluates eight cognitive domains: attention-concentration, executive function, memory, language, visual construction skills, abstract thinking, calculation, and orientation [45]. Higher scores indicate better cognitive function; the maximum possible MoCA score is 30 while scores below 21 suggest cognitive impairment. The adapted language version of MoCA was used in this study [46]. The research physiotherapist received specific training for MoCA test administration from a neurologist in the course of a cognitive rehabilitation training course. The Montreal Cognitive Assessment (MoCA) demonstrated good test-retest reliability, with an ICC of 0.64 for the Total Score and fair to poor reliability for the Memory Index Score, ranging from ICC 0.32 to 0.48 [47].

### Statistical analysis

Descriptive statistics were expressed as mean ± standard deviation (s.d.) values for normally distributed parametric data, as median with minimum-maximum values otherwise. Pearson’s Chi-Square and Fisher’s Exact tests were used to compare the differences between categorical variables. Shapiro-Wilk test was used as a test of normality to determine whether parametric data conformed to Gaussian normal distribution (*https*:*//www*.*statskingdom*.*com*). For categorical data, set numbers and their percentages were given. For normally distributed data, Independent Samples t-test was used to compare inter-group (Group-I = AOT-E and Group-II = E) differences while Paired Samples t-test was used for intra-group pre- and post-intervention measurements. For data failing the normality test, Mann-Whitney U-test and Wilcoxon test were used respectively for these comparisons. Probability (p) value ≤ 0.05 was adopted as the statistical significance decision level. Statistical analysis utilized Jamovi (Jamovi Project, version 2.2.5.0, 2022, *https*:*//www*.*jamovi*.*org*) and JASP (Jeffreys’ Amazing Statistics Program, version 0.16.1, *https*:*//jasp-stats*.*org*) program packages.

Effect sizes of the improvements observed according to Cohen’s-d statistic were computed as the ratio of the absolute change of the outcome measure to its pooled sd. in the group assessed before and after training. Effect sizes were considered strong if ≥0.8, medium if ≥0.5 and weak otherwise.

### Sample size

The power of the study was calculated using the Gpower package program. (Version 3.1.9.7, 2020 obtained from *https*:*//www*.*psychologie*.*hhu*.*de/arbeitsgruppen/allgemeine-psychologie-und-arbeitspsychologie/gpower*). First, target effect size Cohen’s-d (“d”) was estimated based on the study of Leem et al. using their TUG measurement outcomes which resulted in d = 1.31 and d = 1.22 for action observation and simple exercise groups respectively [30]. The minimum total sample size for the study was consequently determined as 38 for a strong target effect size d = 1.22 with 95% (β = 0.05) power at 95% (α = 0.05) confidence level. However, considering that the dropout rate would be high due to pandemic, it was decided to include at least 60 participants in the study.

## Results

17 participants dropped out the study (6 people with health problems, 11 people attendance discontinuty) A total of 43 participants completed the study and were included in the analysis; their demographic and clinical characteristics are presented in Table 2. There were no differences between the groups in terms of clinical and demographic features except for body weight at the baseline (p>0.05). Although the mean weight of group II was higher, both groups were found to be similar when body mass index was calculated (p<0.05).

**Table 2:** Demographic and clinical characteristics of the study groups.

	Groups		
	Group I	Group II	p-value*
	N = 22	N = 21	
**Age** (year) ^†^	70.3 ± 5.4	69.7 ± 4.1	0.66**
**Sex** ^‡^			
Male	7 (31.8)	4 (19.0)	0.54
Female	15 (68.2)	17 (81.0)	
**Mass** (kg) ^†^	70.1 ± 10.3	78.3 ± 12.9	**0.027** **
**Height** (cm) ^†^	162.5 ± 9.6	165.4 ± 7.6	0.27**
**BMI** (kg/m^2^) ^†^	26.7 ± 4.5	28.7 ± 4.5	0.17**
**BMI groups** ^‡^			
Normal (18.5–24.99 kg/m^2^)	9 (40.9)	4 (19.0)	0.45
Overweight (25–29.99 kg/m^2^)	7 (31.8)	8 (38.1)	
Obese class I (30–34.99 kg/m^2^)	5 (22.7)	8 (38.1)	
Obese class II (35–39.99 kg/m^2^)	1 (4.5)	1 (4.8)	
**Marital statu**s			
Married	12 (54.5)	10 (47.6)	0.88
Single/divorced/widowed	10 (45.5)	11 (52.4)	
**Education status** ^‡^			
Primary	10 (45.5)	7 (33.3)	0.70
College	6 (27.3)	6 (28.6)	
University	6 (27.3)	8 (38.1)	
**Employment** ^‡^			
Unemployed	8 (36.4)	10 (47.6)	0.66
Retired	14 (63.6)	11 (52.4)	
**Alcohol consumption** ^‡^	7 (31.8)	4 (19.0)	0.54
**Smoking** ^‡^	9 (40.9)	5 (23.8)	0.38
**Living in** ^‡^			
Apartment flat	19 (86.4)	19 (90.5)	0.999
Self-contained flat	3 (13.6)	2 (9.5)	
**Comorbidities** ^‡^			
Hypertension	12 (54.5)	12 (57.1)	0.999
Diabetes mellitus	11 (50.0)	4 (19.0)	0.070
Osteoarthritis	12 (54.5)	12 (57.1)	0.999
Hyperlipidemia	12 (54.5)	11 (52.4)	0.999
**Physical activity status** ^‡^	‐‐	‐‐	
High (>3000 MET-min/week)			
Moderate (600–3000 MET-min/week)	6 (27.3)	9 (42.9)	0.45
Low (<600 MET-min/week)	16 (72.7)	12 (57.1)	
**International Physical Activity Questionnaire (IPAQ-SF)**	663 ± 350 ^†^	775 ± 342 ^†^	0.29**
**Number of falling within the last one year**	1.0 [0.0–5.0] ^§^	1.0 [0.0–5.0] ^§^	0.72***

*Note*. ^†^: mean ± standard deviation

^§^: median [min-max]

^‡^: n (%)

AOT-E: Action observation therapy plus exercise, E: exercise only, BMI: body mass index.

*: Pearson Chi-Square, Fisher’s Exact or Fisher Freeman Halton test.

**: Independent Samples T-Test.

***: Mann-Whitney U test.

Approximately 75% of all participants were female (N = 33/43), mean age was 70 ± 4.8 years, and 30% were of normal weight (BMI≤25.) There was no one with a high level of physical activity (>3000 MET min/week) in either group, the majority had low levels of physical activity (<600 MET-min/week, 73% in Group-I, 57% in Group-II) according to IPAQ-SF evaluation guidelines. The number of falls in the last year was reported between 0 to 5 times in both groups, the number of participants who did not fall was determined as eight in Group-I, and seven in Group-II.

The results showing the effect of the exercise program are shown in Table 3. It was found that the baseline values of both groups were all similar in terms of strength, balance, mobility and cognitive performance (5XSST, TBGA, ABC, TUG and MoCA), and the groups showed a homogeneous distribution (p>0.05). Based on the normative values of this test reported for healthy older adults, all of our participants were found to have low functional strength (>12s) at baseline. After the intervention, there was a very significant improvement in all outcome measures in both groups (all p<0.02). In the analysis of the differences between groups, it was found that the AOT group progress was significantly better for all outcomes except MoCA (p<0.05). The effect sizes for all test outcomes, as measured by Cohen’s-d statistic, were computed considerably stronger as well in AOT group except for MoCA.

**Table 3:** Assessment measures of the participants before (pre-I) and after the interventions (post-I).

		Groups		
		Group I	Group II	p-value*
		N = 22	N = 21	
**Montreal Cognitive Assessment scale** ^**†**^	Pre-I	23.5 ± 2.50	23.6 ± 2.27	0.87
		23 [21 – 28] ^§^	24 [21 – 28] ^§^	
	Post-I	24.0 ± 2.75	24.0 ± 2.28	
		23 [21 – 28] ^§^	24 [21 – 28] ^§^	
	*Δ*	0.55 ± 0.74	0.38 ± 0.59	
		0 [0 – 2] ^§^	0 [0 – 2] ^§^	0.63**
	*p* ****	**0.009**	**0.014**	
	*Cohen’s-d*	0.15	0.12	
**Timed up and go test (sec)** ^**†**^	Pre-I	12.6 ± 1.64	12.2 ± 2.18	0.50
	Post-I	10.7 ± 1.75	11.3 ± 2.23	
	*Δ*	-1.98 ± 1.04	-0.98 ± 0.31	**<0.0005**
	*p* **	**<0.0001**	**<0.0001**	
	*Cohen’s-d*	**0.83**	0.31	
**Five Times-Sit-to-Stand test (sec)** ^**†**^	Pre-I	13.1 ± 1.62	13.5 ± 2.48	0.51
	Post-I	11.1 ± 1.90	12.6 ± 2.49	
	*Δ*	-1.92 ± 0.88	-0.85 ± 0.31	**<0.0001**
	p**	**<0.0001**	**<0.0001**	
	*Cohen’s-d*	**0.77**	0.24	
**Tinetti Balance and Gait Scale test/balance** ^**†**^	Pre-I	9.59 ± 2.09	9.67 ± 2.42	0.91
	Post-I	12.4 ± 2.24	11.05 ± 2.33	
	*Δ*	2.77 ± 0.92	1.38 ± 0.67	**<0.0001**
	p**	**<0.0001**	**<0.0001**	
	*Cohen’s-d*	**0.91**	0.41	
**Tinetti Balance and Gait Scale test /walking** ^**†**^	Pre-I	7.59 ± 1.18	8.14 ± 1.31	0.15
	Post-I	8.73 ± 1.45	8.67 ± 1.39	
	*Δ*	1.14 ± 0.83	0.52 ± 0.51	**<0.01**
	p**	**<0.0001**	**<0.0001**	
	*Cohen’s-d*	*0*.*61*	0.27	
**Tinetti Balance and Gait Scale test/total** ^**†**^	Pre-I	17.2 ± 2.77	17.8 ± 3.47	0.51
	Post-I	21.1 ± 3.45	19.7 ± 3.47	
	*Δ*	3.91 ± 1.31	1.90 ± 0.70	**<0.0001**
	p**	**<0.0001**	**<0.0001**	
	*Cohen’s-d*	**0.88**	0.39	
**Activity-Specific Balance Confidence scale score** ^**†**^	Pre-I	56.4 ± 12.55	58.6 ± 15.41	0.61
	Post-I	69.1 ± 15.37	64.4 ± 16.18	
	*Δ*	12.7 ± 5.60	5.77 ± 3.05	**<0.0001**
	p**	**<0.0001**	**<0.0001**	
	*Cohen’s-d*	*0*.*64*	0.26	

^†^: mean ± standard deviation

^§^: median [min-max], *Δ*: Difference = (Post-I)–(Pre-I)

AOT-E: Action observation therapy plus exercise, E: exercise only.

*: Independent Samples T-Test.

**: Paired Samples T-test.

***: Mann-Whitney U-test.

****: Wilcoxon test.

## Discussion

In this study, we investigated the effects of a community-based, structured exercise program combined with action observation on strength, balance, mobility, and cognition in healthy older adults who were socially isolated during the pandemic. We applied a combined exercise program with Action Observation Training (AOT) to one group, and exercise alone to the other group and compared the effect of both programs. We observed significant improvements in all outcome measurement results in both AOT and simple exercise groups after the intervention, thus confirming the effectiveness of our community-based training method. Comparing post-intervention improvements between groups, we further found that the progress effect size in the group with AOT was significantly stronger in all outcome measures, except for cognitive abilities (MoCA). Thus, our hypothesis that AOT would increase the effectiveness of the exercise program was partly confirmed. We used the Montreal Cognitive Assessment (MoCA) as our assessment tool for evaluating cognitive function in the study participants. The MoCA is a widely recognized instrument known for its sensitivity in detecting cognitive changes, particularly in individuals with mild cognitive impairment or early stages of dementia. However, it’s important to acknowledge that MoCA’s sensitivity can be influenced by various factors, including practice effects, which may impact the interpretation of scores when used as an outcome measure in repeated assessments.

The study’s context is crucial in understanding the challenges and potential impact of our interventions. It has been demonstrated that older adults’ physical activity levels correlate strongly with the time they spend outdoors [48]. However, the COVID-19 pandemic imposed severe restrictions on outdoor activities, leading to a notable increase in sedentary behavior and inactivity, particularly among older adults. It’s noteworthy that the majority of our participants exhibited low levels of physical activity, as indicated by their baseline IPAQ-SF scores, which were less than 600 MET-min/week. We refrained from reassessing physical activity levels at the study’s conclusion since the IPAQ-SF evaluates activity for the preceding week, and questioning it immediately after the program’s completion could introduce bias.

In light of previous research findings, community-based interventions tailored to older adults and incorporating face-to-face or group sessions tend to yield greater effectiveness [49]. Additionally, shorter intervention periods, such as 10 weeks, have been suggested to enhance compliance. In our study, we implemented group exercises specifically designed to address the strength, balance, and mobility aspects of physical activity that are particularly relevant to older adults. These sessions occurred three times a week for eight weeks, totaling 24 sessions. Despite the challenges posed by the pandemic, only 28% of the participants were unable to continue their participation. Those who persisted successfully completed all the sessions. Notably, the group sizes for exercise sessions averaged between 5 to 7 individuals during the final weeks of training, indicating a high level of compliance, similar to what has been observed in previous studies.

A step-reduction study has shed light on the detrimental effects of short-term (14 days) physical inactivity on both the metabolic and musculoskeletal systems [50]. In our investigation, we employed the Five Times Sit to Stand test (5XSST) to assess the functional strength of the lower extremities in our participants. Our baseline assessments revealed that, based on normative values for healthy older adults, all of our subjects exhibited low functional strength (>12 seconds). Although age-related declines in muscle strength are expected, none of our participants initially reached the anticipated healthy values. This observation may be attributed to prolonged periods of inactivity during the pandemic. However, it is noteworthy that after participating in our exercise training, the functional strength of the lower extremities significantly increased in both our study groups, ultimately reaching the expected normative values. This outcome underscores the effectiveness of our exercise training methods in enhancing muscle strength.

Drawing parallels to a similar study by Leem et al. involving older adults, where the effects of action observation (lasting 20 minutes) and simple Otago exercises (comprising strength and balance exercises) were compared, our findings align with prior research. Despite the shorter total number of sessions in our study (24 sessions), Group I, which received AOT, experienced a more substantial increase in muscle strength. This improvement was not only statistically significant but also clinically more pronounced, as indicated by the effect size Cohen’s-d (1.09 for Group I vs. 0.34 for Group II). These results provide further support for previous evidence suggesting that the action observation technique accelerates motor learning and enhances motor skills, leading to improved muscle strength [30].

The impact of Action Observation Therapy (AOT) on balance performance in healthy populations has been a subject of investigation in previous studies. For instance, Gatti et al. conducted research on 79 healthy young adults, dividing them into action observation, action observation plus imitation, balance training, and control groups. These groups underwent daily 30-minute training sessions over three weeks, and posturography was used for evaluation. Their findings indicated that both action observation plus imitation and balance training had comparable effects in enhancing postural control, with even action observation alone showing a tendency to improve postural control [46]. Additionally, Leem et al., in a study involving older adults, suggested that exercises combined with AOT were more effective in enhancing dynamic balance and fall efficiency [30].

In our study, the mean baseline scores of our participants on the Tinetti test, which quantifies balance, indicated a high risk of falling (≤18 points). However, our results demonstrate the effectiveness of the exercise program we implemented in improving the participants’ balance skills. Following the training, we observed significant enhancements in the balance, walking, and total scores of the Tinetti test in both study groups, resulting in a reduction of the risk of falling to an intermediate level (19–23 points). Furthermore, when comparing post-intervention improvements between the groups, we noted that the progress measured in AOT Group-I was significantly greater in terms of all three Tinetti test scores (balance, walking, and total). Notably, the effect of this change was clinically more pronounced (Cohen’s-d: 1.28, 0.86, 1.25, respectively for Group-I vs. 0.58, 0.39, 0.55 for Group-II). Additionally, the self-reported balance confidence scale (ABC test) showed a baseline mean value for our study participants indicative of a low functional status (<60%). Nevertheless, after the training, it significantly improved in both groups, surpassing the threshold value. This improvement in effect size was also notably stronger in the AOT Group-I (Cohen’s-d 0.91 for Group-I vs. 0.37 for Group-II). These findings are of paramount importance as they underscore the enhancement of exercise training benefits by action observation therapy, particularly in improving multidimensional skills such as balance.

We selected the Timed Up and Go (TUG) test to assess the mobility of our participants, a test that is positively correlated with age. Normatively, it is expected to be approximately 9.4 seconds for individuals over 65 years of age [51]. At the outset, our participants demonstrated relatively low baseline performance, exceeding the critical threshold indicating a risk of falling (>12 seconds). In a previous study, despite older participants with lower TUG scores (80 years, 15–16 seconds) compared to ours, the most significant improvement was observed in the combined Action Observation Therapy (AOT) group [30]. Another study examined the effects of action observation on mobility and motor performance in individuals with a mean age of 70.5±6.4 years. It reported improved walking speed and enhanced movement control in dual tasks after 10 sessions of AOT [29]. In our study, TUG mobility scores significantly improved in both groups after training, with a more noticeable effect in our AOT Group-I. These results highlight the effectiveness of AOT combined with exercise in enhancing mobility among older adults, consistent with previous research findings. Additionally, the TUG test, selected as our primary outcome measure, demonstrated substantial improvement, further confirming the robustness of our findings and the confidence level in our results.

In our study, we aimed to investigate the potential cognitive benefits of a multicomponent program that included community-based outdoor activities, group exercise, social participation, and the mental strategy of action observation. We employed the Montreal Cognitive Assessment (MoCA) to evaluate cognitive function among participants. At baseline, none of our participants exhibited cognitive impairment, as indicated by MoCA scores ≥21 points. However, none of them achieved a perfect score, suggesting room for improvement in cognitive function (maximum observed score: 28<30). Upon completion of our study, both groups showed small but statistically significant improvements in MoCA assessments. However, there was no significant difference between the groups favoring the Action Observation Therapy (AOT) group. Furthermore, the observed improvements, though statistically significant, were of minimal clinical significance, with effect sizes (Cohen’s-d) below 0.2. Surprisingly, our exercise program, even with the inclusion of AOT, did not yield substantial enhancements in cognitive abilities. Several factors may explain these results. First, our participants had cognitive reserves within the normal range, which might have limited the potential for cognitive improvement. Additionally, the relatively low exercise intensity and the emphasis on cognitive task training during our sessions could have contributed to these findings.

There is limited existing literature on the effects of AOT on cognitive functions, with only one trial specifically examining this aspect. In that study, the combination of action observation and gait training was investigated in individuals with mild cognitive impairment, reporting better progress in MoCA scores and improved cognition along with enhanced walking performance [52]. These disparate findings underscore the need for further research in this area to better understand the potential impact of AOT on cognitive functions.

### Study limitations

Our study has certain limitations that need to be acknowledged. First, the absence of a placebo group, where no exercise program was applied, might be considered a potential weakness. However, we believe that ethically, it was not appropriate to deprive older adults of the opportunity to engage in exercise, especially during a pandemic with restrictive conditions that limited their outdoor activities. Second, we were unable to assess the long-term effects of the exercise program, as our study focused on short-term outcomes.

Additionally, we recognize another limitation related to the blinding of outcome assessors. We did not implement blinding procedures for the individuals responsible for assessing the outcomes. This non-blinding of outcome assessors could be considered a limitation of our study and should be taken into account when interpreting the results.

It should be noted that the participants in this study were recruited from an upper-middle-class metropolitan urban district. While this district provided practical advantages for conducting the research, such as access to resources and facilities, it may limit the generalizability of our findings to populations from diverse socioeconomic backgrounds or rural areas. The demographic characteristics of the study participants could influence the applicability of the results to broader populations. Future research endeavors should strive to include a more diverse participant sample to enhance the external validity and generalizability of the study’s conclusions.

Besides, it should be noted that our study predominantly consisted of female participants. While this allowed us to investigate the effects of our intervention within this specific demographic, it may limit the generalizability of our findings to a more diverse population. Furthermore, research on potential sex and gender differences in the effects of Action Observation Therapy (AOT) remains relatively limited in the existing literature. This aspect warrants further exploration in future studies to enhance our understanding of how AOT may impact various demographic groups.

## Conclusions

The results of this study showed that community-based group exercises have positive effects in older adults whose potential health risks are increased due to social isolation and reduced participation in physical activity during the pandemic period. The combined effect of the exercises with the added Action Observation Therapy showed that it was a more effective program than the exercises alone. It would be beneficial to add a simple, practical and economical method such as action observation, which strengthens motor learning to any exercise program aimed at improving health in the older adult population.
